# Characterization and subcellular localization of histone deacetylases and their roles in response to abiotic stresses in soybean

**DOI:** 10.1186/s12870-018-1454-7

**Published:** 2018-10-11

**Authors:** Chao Yang, Wenjin Shen, Hongfeng Chen, Liutian Chu, Yingchao Xu, Xiaochen Zhou, Chuanliang Liu, Chunmiao Chen, Jiahui Zeng, Jin Liu, Qianfeng Li, Caiji Gao, Jean-Benoit Charron, Ming Luo

**Affiliations:** 1 Guangdong Provincial Key Laboratory of Applied Botany, Key Laboratory of South China Agricultural Plant Molecular Analysis and Genetic Improvement, South China Botanical Garden, Chinese Academy of Sciences, Guangzhou, 510650 China; 2 Guangdong Provincial Key Laboratory of Biotechnology for Plant Development, School of Life Sciences, South China Normal University, Guangzhou, 510631 China; 3 University of Chinese Academy of Sciences, Beijing, 100049 China; 4 Institute for Food and Bioresource Engineering, Department of Energy and Resources Engineering and BIC-ESAT, College of Engineering, Peking University, Beijing, 100871 China; 5 Key Laboratory of Crop Genetics and Physiology of Jiangsu Province/Key Laboratory of Plant Functional Genomics of the Ministry of Education, College of Agriculture, Yangzhou University, Yangzhou, 225009 China; 6 Department of Plant Science, McGill University, Sainte-Anne-de-Bellevue, QC, Canada

**Keywords:** Histone deacetylases, Subcellular localization, Gene expression, Abiotic stresses, Soybean

## Abstract

**Background:**

Histone deacetylases (HDACs) function as key epigenetic factors in repressing the expression of genes in multiple aspects of plant growth, development and plant response to abiotic or biotic stresses. To date, the molecular function of HDACs is well described in *Arabidopsis thaliana*, but no systematic analysis of this gene family in soybean (*Glycine max*) has been reported.

**Results:**

In this study, 28 *HDAC* genes from soybean genome were identified, which were asymmetrically distributed on 12 chromosomes. Phylogenetic analysis demonstrated that GmHDACs fall into three major groups previously named RPD3/HDA1, SIR2, and HD2. Subcellular localization analysis revealed that YFP-tagged GmSRT4, GmHDT2 and GmHDT4 were predominantly localized in the nucleus, whereas GmHDA6, GmHDA13, GmHDA14 and GmHDA16 were found in both the cytoplasm and nucleus. Real-time quantitative PCR showed that *GmHDA6*, *GmHDA13*, *GmHDA14*, *GmHDA16* and *GmHDT4* were broadly expressed across plant tissues, while *GmHDA8, GmSRT2*, *GmSRT4* and *GmHDT2* showed differential expression across various tissues. Interestingly, we measured differential changes in *GmHDACs* transcripts accumulation in response to several abiotic cues, indicating that these epigenetic modifiers could potentially be part of a dynamic transcriptional response to stress in soybean. Finally, we show that the levels of histone marks previously reported to be associated with plant HDACs are modulated by cold and heat in this legume.

**Conclusion:**

We have identified and classified 28 *HDAC* genes in soybean. Our data provides insights into the evolution of the *HDAC* gene family and further support the hypothesis that these genes are important for the plant responses to environmental stress.

**Electronic supplementary material:**

The online version of this article (10.1186/s12870-018-1454-7) contains supplementary material, which is available to authorized users.

## Background

Throughout their life course, plants are frequently exposed to suboptimal environmental conditions that cause adverse effects on their growth and development. Abiotic stress is one of the major causes of agricultural losses in the world [1, 2]. Soybean (*Glycine max*) is an important global crop desirable for its high protein content and oil. More than 50% of globally consumed edible oil is contributed by soybeans while the proteins of this legume are highly desirable for food and feed applications [3]. However, the production of soybeans is greatly affected by abiotic stresses such as salt, drought, cold, heat, water submergence and heavy metals [3]. Recently, studies have demonstrated that epigenetic processes play vital regulatory roles in plant abiotic stress responses [4–7]. Chromatin structure and gene expression changes in response to abiotic stress are controlled through epigenetic mechanisms such as histone modification and chromatin remodeling. Among them, histone acetylation is regulated by histone acetyltransferases (HATs) and histone deacetylases (HDACs).

In the past decades, a large number of HDACs have been identified and characterized in plants, which can be grouped into three different families: the RPD3/HDA1, the SIR2, and the HD2. Members of the RPD3/HDA1 and the SIR2 families are proteins homologous to the yeast Reduced Potassium Dependency 3 (RPD3)/HDA1 and Silent Information Regulator 2 (SIR2), respectively, whereas the HD2 family was originally characterized in maize and appears to be present only in plants [8–10]. Members of the SIR2 family have a catalytic domain that requires nicotine adenine dinucleotide (NAD) as a cofactor [11], while members of the RPD3/HDA1 family share HDAC domain sequence homology and require a Zn^2+^ cofactor for deacetylase activity [9]. The HD2 family proteins contain a conserved pentapeptide motif (MEFWG) at the N-terminus and are considered to be zinc-dependent HDACs [12].

The *Arabidopsis* HDACs have been well characterized. The genome of this model plant encodes 18 HDACs distributed into the three aforementioned HDAC families [10, 13]. Increasing evidence suggests that AtHDACs play essential roles in regulating multiple aspects of plant growth and development [4, 14, 15]. AtHDA6 is a member of the RPD3/HDA1 family that was originally reported to play an important role in coordinating downstream gene silencing and maintenance of DNA methylation [16, 17]. It was also demonstrated that HDA6 interacted with a number of protein partners, such as FLOWERING LOCUS D (FLD), ASYMMETRIC LEAVES 1 (AS1), TOPLESS (TPL), and JASMONATE ZIM-DOMAIN (JAZ) to regulate flowering, leaf development, circadian transcription and hormonal responses by modulating the transcription of their respective target genes through histone deacetylation [18–21]. Other members of the RDP3/HDA1 family, such as AtHDA5, AtHDA7, AtHDA9, AtHDA14, AtHDA15, AtHDA18 and AtHDA19, were also reported to play important roles in plant growth and development [22–37]. For example, *hda19* mutants displayed various developmental abnormalities, such as early senescence, suppression of apical dominance, flower defects, and male and female sterility, indicative of the importance of AtHDA19 for proper vegetative development [22, 24, 31]. HDA5 forms a protein complex with HDA6, FLD and MSI1-like WD40 repeat 4 (FVE/MSI4) in regulation of flowering time by repressing *FLOWERING LOCUS C* (*FLC*) expression through histone deacetylation [34]. The BRI1-EMS-SUPPRESSOR1 (BES1)-TPL-HDA19 repressor complex is required for deacetylation of *ABA-insensitive 3* (*ABI3*) gene in controlling early seedling development [32]. Additionally, members of the HD2 family play crucial roles in plant development [38–42]. For instance, silencing of *HD2A* in *Arabidopsis* resulted in aborted seed development [38], while overexpression of *HD2A* caused morphological defects of leaves and flowers, delayed flowering and aborted seed development [39]. It was demonstrated that the SIR2 family protein AtSRT2 plays a negative role in plant basal defense against the pathogen PstDC3000, and the expression of *AtSRT2* was repressed in response to the pathogen infection [43]*.* In rice (*Oryza sativa*), OsSRT1 is required for repressing the expression of starch metabolism genes during seed development [44].

In *Arabidopsis*, AtHDACs play vital roles in plant responses to abiotic and biotic stresses [4, 45–53]. It was reported that *AtHDACs* were responsive to various environmental cues at the transcription level [13], for example, *AtHDA6* and *AtHDA19* are induced by JA treatment [45], whereas the expression of *HD2A*, *HD2B*, *HD2C*, and *HD2D* is repressed by ABA and NaCl [49, 54]. *AtHDA9* was shown to negatively regulate high salt and drought responses [53]. On the other hand, *AtHDA6*, *AtHD2C*, and *AtHD2D* confer plant resistance under high salt, drought, cold or freezing conditions [49–51, 55]. Recently, emerging evidence indicates that *hda19* plants exhibit tolerance to high salinity stress, while *hda5/14/15/18* plants exhibit hypersensitivity to salt stress. This suggests that AtHDA19 and AtHDA5/14/15/18 control plant responses to salt stress in different pathways [53]. While, different members of the HDAC family have been found to mediate distinct aspects of the plant growth and development as well as its abiotic and biotic stress responses, the characterization of *HDAC* genes in soybean has not been reported yet.

In this study, we have identified 28 *HDAC* coding genes in the soybean genome. YFP-tagged transient expression assays confirmed the subcellular localization of GmHDACs. While tissue-specific and stress-responsive expression patterns for nine representative genes were determined using quantitative RT-PCR. In addition, the levels of histone acetylation and methylation were analyzed under cold and heat treatments. Together, our results shed light on the involvement of *GmHDAC* genes in various aspects of plant growth and development including the response to abiotic stress.

## Results

### Identification and phylogenetic analysis of soybean histone deacetylases

The protein sequences of AtHDACs were used as queries to conduct sequence homology searches against the Soybase database (https://soybase.org/). In total, 28 independent soybean GmHDACs were identified. (Table 1). The complete open reading frames (ORFs) of the retrieved *GmHDAC* genes ranged from 582 to 1971 bp, while predicted proteins ranged from 193 to 656 amino acids with calculated molecular weights from 21.37 to 72.99 kDa and isoelectric points from 4.29 to 9.40 (Table 1). Further bioinformatics analyses indicated that GmHDACs were potentially localized in several organelles such as the nucleus, cytoplasm, chloroplast and mitochondria (Table 1).

**Table 1:** Overview of histone deacetylases genes identified in soybean

Gene ID			DNA attributes			Protein attributes			
Gene name^a^	Gene locus^b^	Accession number^c^	Chromosome	ORF (bp)	No. of exons	Length (aa)	MW (kDa)	PI	Localization^d^
GmHDA1	Glyma.01 g245100	XP_003517607.1	1	1494	7	497	56.28	5.30	nucl, cyto
GmHDA2	Glyma.04 g000200	XP_003543935.1	4	1494	7	497	55.96	5.04	cyto, nucl
GmHDA3	Glyma.04 g187000	XP_006578653.1	4	1398	6	465	52.18	5.28	cyto, chlo
GmHDA4	Glyma.04 g187100	KRH63610.1	4	594	2	197	22.26	6.31	cyto
GmHDA5	Glyma.05 g012900	KRH56687.1	5	1254	14	417	45.28	7.39	nucl, cyto
GmHDA6	Glyma.05 g021400	XP_003524633.1	5	1971	14	656	73.04	5.25	cyto, nucl
GmHDA7	Glyma.05 g040600	XP_003525556.1	5	1431	6	476	53.30	5.24	nucl
GmHDA8	Glyma.05 g192600	XP_014631275.1	5	1263	9	420	45.54	6.09	chlo, nucl
GmHDA9	Glyma.06 g000100	XP_003526730.1	6	1494	7	497	55.95	5.06	nucl, cyto
GmHDA10	Glyma.06 g178700	KRH54335.1	6	705	1	234	26.74	8.88	chlo, cyto
GmHDA11	Glyma.11 g000300	XP_006590384.1	11	1494	7	497	56.12	5.22	nucl, cyto
GmHDA12	Glyma.11 g187800	XP_003538135.1	11	1290	14	429	48.94	4.98	cyto, nucl
GmHDA13	Glyma.12 g086700	XP_003539814.1	12	1290	14	429	48.94	5.06	cyto, mito
GmHDA14	Glyma.12 g188200	XP_003540263.1	12	1146	3	381	41.18	5.44	cyto, chlo
GmHDA15	Glyma.17 g078000	KRH03123.1	17	1971	14	656	72.99	5.35	cyto, nucl
GmHDA16	Glyma.17 g085700	XP_003549603.1	17	1419	6	472	52.92	5.26	nucl, chlo
GmHDA17	Glyma.17 g120900	XP_006600776.1	17	1632	17	543	59.6	5.91	nucl, cyto
GmHDA18	Glyma.17 g229600	XP_003550277.1	17	1047	13	348	38.50	6.29	cyto, nucl
GmSRT1	Glyma.04 g210000	XP_003522478.1	4	1182	11	393	43.46	9.40	chlo, mito
GmSRT2	Glyma.06 g156000	XP_003528059.2	6	1179	11	392	43.21	9.32	chlo, mito
GmSRT3	Glyma.08 g330200	KHN11152.1	8	1302	13	433	48.09	9.11	chlo, nucl
GmSRT4	Glyma.18g076300	XP_003551434.1	18	1440	14	479	53.19	9.15	nucl, cyto
GmHDT1	Glyma.03 g190700	NP_001240859.1	3	867	10	288	31.46	4.64	nucl
GmHDT2	Glyma.11 g189500	XP_006591094.1	11	870	9	289	30.77	4.80	nucl
GmHDT3	Glyma.12 g084700	KRH25160.1	12	900	9	299	31.77	4.75	nucl
GmHDT4	Glyma.12 g181400	KRH26575.1	12	924	10	307	33.31	4.9	nucl
GmHDT5	Glyma.13 g319500	KRH22734.1	13	582	6	193	21.37	4.29	chlo, cyto
GmHDT6	Glyma.19 g191000	XP_003554417.1	19	882	9	293	31.91	4.61	nucl

^a^Systematic designation given to soybean histone deacetylase genes

^b^Accession number of Soybase (http://soybase.org/) locus ID

^c^Accession numbers of protein sequence available at NCBI (http:// www.ncbi.nlm.nih.gov/)

^d^Subcellular Localization of soybean histone deacetylases supported by WoLF PSORT (http://www.genscript.com/psort/wolf_psort.html)

CDS, coding sequence; No., number; MW, molecular weight; PI, isoelectric point

Nuc, nuclear; Cyto, cytoplasm; Chl, chloroplast; Mito, mitochondrion

**Fig. 1:**
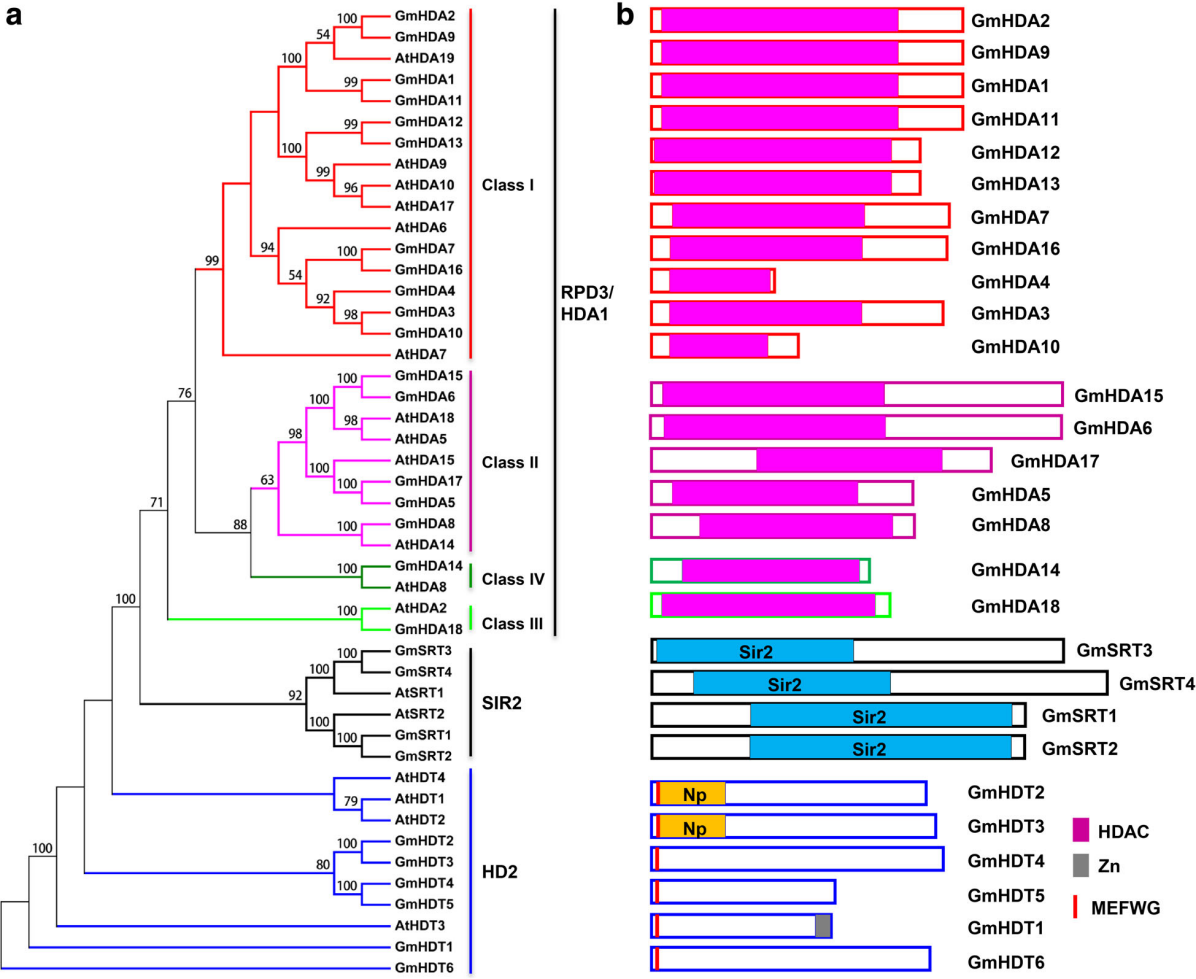
Phylogenetic tree and domain architecture of HDACs in soybean. **a** Phylogenetic tree of HDAC proteins in soybean and *Arabidopsis*. The phylogenetic tree was generated using the MEGA 5.0 software and the Maximum Parsimony method with the following parameters: bootstrap analysis of 1000 replicates and partial deletion. The numbers at the nodes indicate the bootstrap values. **b** The schematic diagrams show the domain organization of these proteins according to analysis by NCBI Batch-CD, SMART and PFAM searches. Different domains are indicated by the use of different colors. The proteins belonging to each family are grouped together

To evaluate the evolutionary relationship of plant histone deacetylases, a phylogenetic analysis was performed using the protein sequences of HDACs from soybean and *Arabidopsis.* The phylogenetic tree indicated that the 28 newly uncovered soybean HDACs are grouped into three types characterized by distinctive protein structures (Table 1 and Fig. 1). In soybean, type I (RPD3/HDA1 family) HDACs consist of 18 members, named GmHDA1 to GmHDA18 based on their coordinates on soybean chromosome (Table 1). All 18 members of this type have a characteristic histone deacetylase domain (Interpro: IPR003084) (Fig. 1b) and can be further divided into four classes based on sequence similarity (Fig. 1a). Class I encompassed 11 GmHDACs, Class II included 5 members while classes III and IV each contained one GmHDAC (Fig. 1a). The phylogenetic analysis also demonstrated that soybean has four type II (SIR2 family) HDACs with highly conserved Sir2 domains (Table 1 and Fig. 1). Finally, six plant-specific HDACs (type III: HD2 family) with conserved N-terminal MEFWG amino acid regions (Fig. 1b), which is required for transcriptional repression followed by a central acidic region rich in glutamic and/or aspartic acid [9] were identified in soybean (Table 1 and Fig. 1). A C2H2 zinc finger domain in the C-terminus portion of GmHDT1 was also detected, which might indicate that this protein has high DNA-binding affinity or could mediate protein-protein interactions. Additionally, both GmHDT2 and GmHDT3 may bind to nucleoplasmins through their N-terminal nucleophosmin domains (Fig. 1b).

### Chromosomal localization and duplications of *HDAC* genes in soybean

**Fig. 2:**
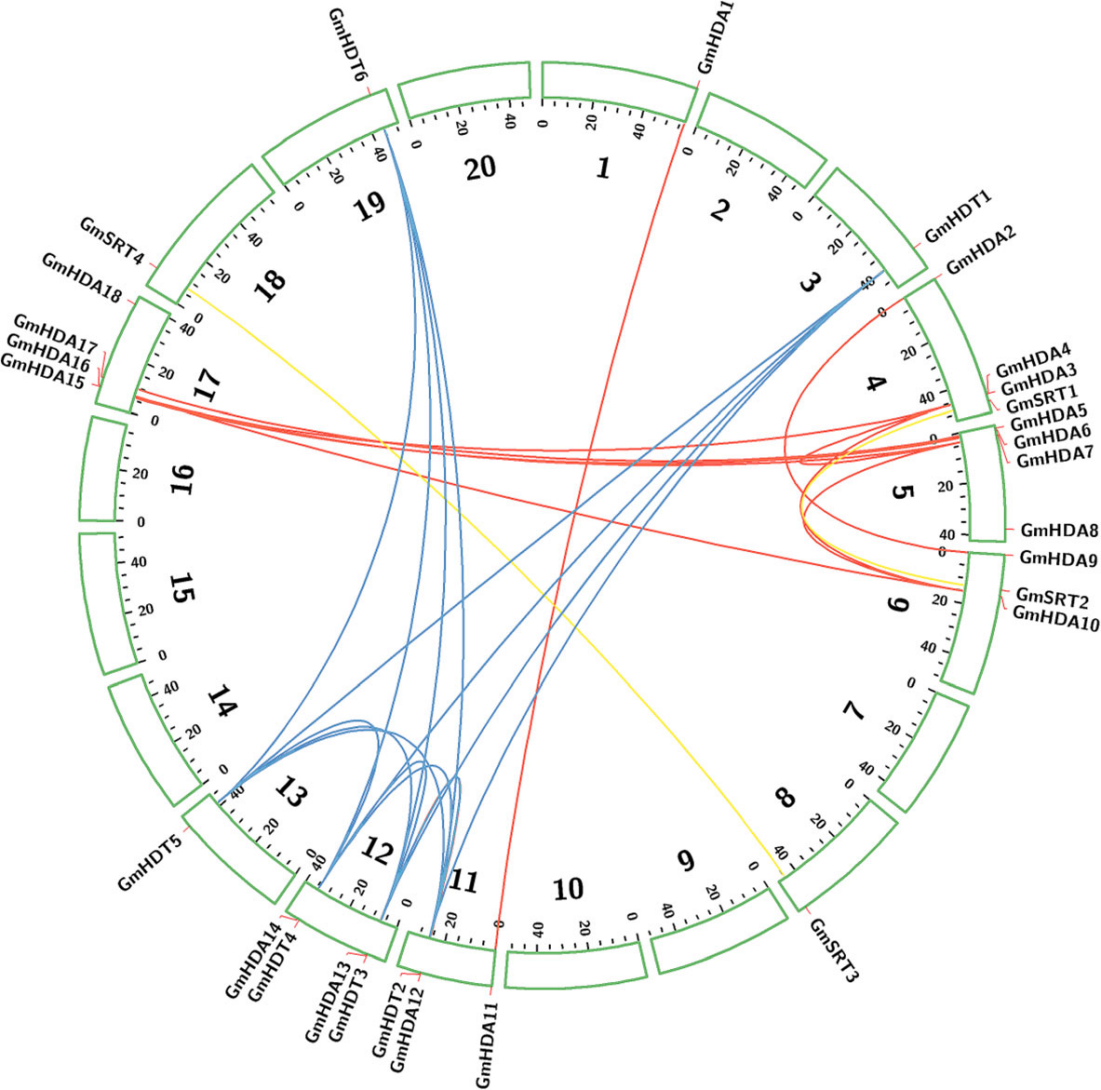
Chromosomal localization and duplication of HDAC genes in soybean. Each box refers to a chromosome, and chromosome numbers are shown beside each chromosome. The scale is in megabase. The approximate location of each soybean HDAC gene is indicated by a short orange line. Colored lines in the circle indicate the linkage group with segmentally duplicated HDAC gene pairs, and segmental duplication regions were determined using the Plant Genome Duplication Database

The chromosomal localization of the 28 *GmHDAC* genes was determined by their genomic distributions on soybean chromosomes. The 28 *GmHDAC* genes were asymmetrically distributed on 12 chromosomes (Fig. 2). Chromosome 4, 5, 12 and 17 each contain the largest number of *HDAC* genes with four each, followed by chromosome 6 and 11 with three genes each, whereas only one *HDAC* gene was present on each of chromosomes 1, 3, 8, 13, 18, and 19. No *HDAC* genes were found on chromosome 2, 7, 9, 10, 14, 15, and 20 (Fig. 2). Moreover, the *HDAC* gene density per chromosome was uneven with the highest densities of *HDACs* at proximal regions of chromosomes 5 and 17 and the distal region of chromosome 4 (Fig. 2).

Tandem duplication and segmental genome duplication are major contributors to the generation and maintenance of gene families. If two paralogous genes are physically close together, we conclude that they probably arose through tandem duplication [56]. It was found that only one gene set (*GmHDA3* and *GmHDA4*) was tandemly distributed on chromosome 4 and was separated by 4407 bp. However, only 23.8% and 29.7% of sequence identity at the nucleotide and protein level were respectively observed between them, indicating that *GmHDA3* and *GmHDA4* have not undergone tandem amplification during evolution. We further investigated whether segmental duplications contributed to the expansion of *HDAC* gene family in soybean. It is noteworthy that 27 duplication sets of *HDAC* genes on the same block were identified. These duplicated genes pairs clustered into a discrete clade in the phylogenetic tree and shared a high degree of identity at the protein level within each pair (Figs. 1 and 2, Additional file 1: Table S1). To evaluate the selection mode of the 27 duplicated gene pairs of *HDAC* in soybean, we calculated the nonsynonymous/synonymous substitution ratios (Ka/Ks). According to the literature a Ka/Ks ratio above 1 indicates positive selection while ratios below and equal to 1 respectively indicate purifying and neural selection [57]. As shown in the Additional file 1: Table S1, the Ka/Ks values of all duplicated gene pairs were less than 1, suggesting that these duplicates likely have been subjected to purifying selection.

### Subcellular localization of GmHDACs

**Fig. 3:**
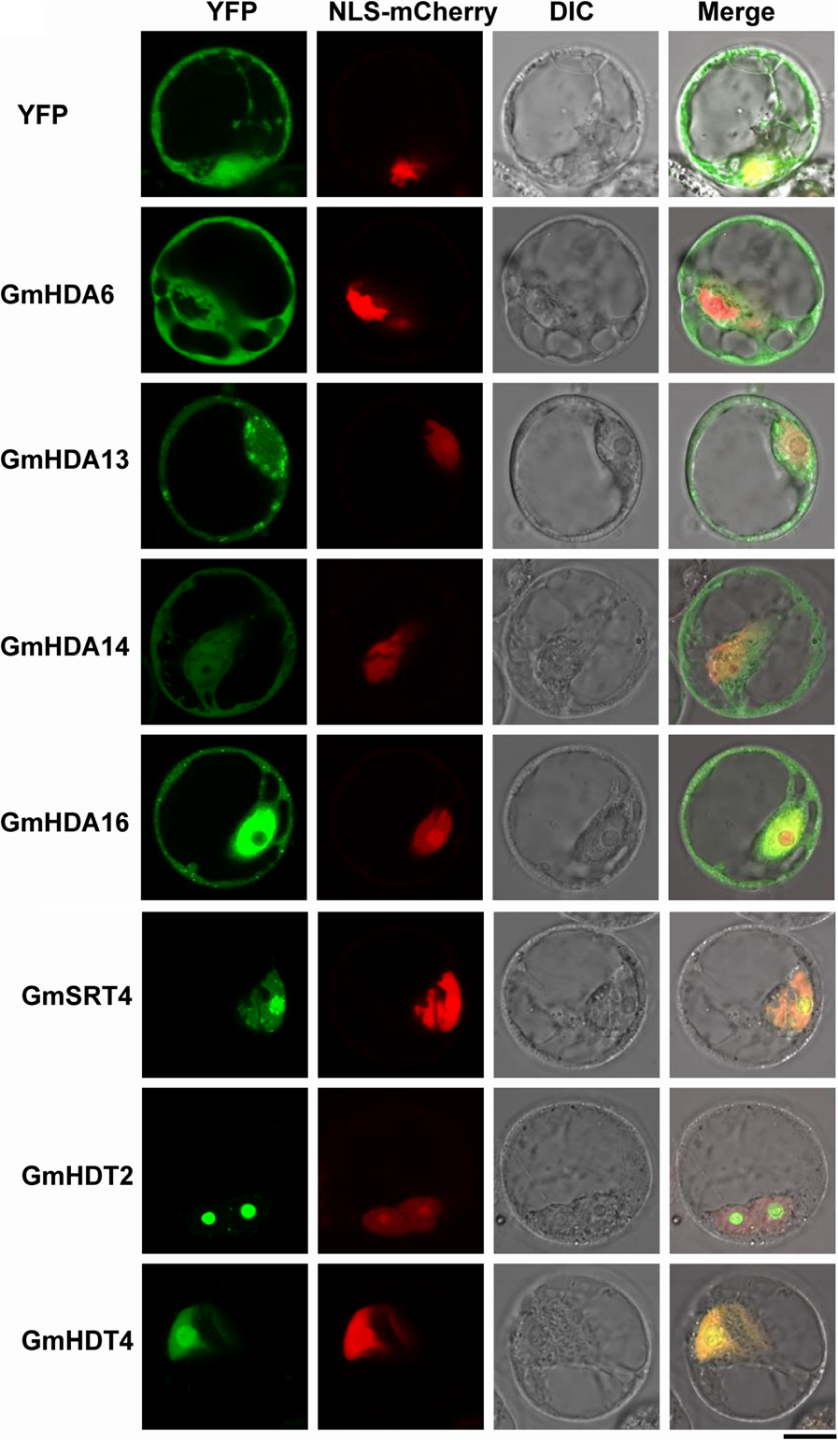
Subcellular localization of GmHDACs. *GmHDACs*-EYFP fusion constructs were used to determine the subcellular localization of GmHDACs. NLS-mCherry was used as a nuclear marker. Fluorescence images of YFP and mCherry were captured with confocal laser scanning microscopy and are shown in green and red, respectively (scale bars, 10 μm)

Prediction analysis indicated that GmHDACs exhibit various patterns of subcellular localization (Table 1). To further determine the subcellular localization of GmHDACs, full-length cDNAs were fused to the *Yellow Fluorescent Protein* (*YFP*) driven by the CaMV 35S promoter and transiently expressed in protoplasts of *Arabidopsis* suspension culture cells. As shown in Fig. 3, four RPD3/HDA1 family members, GmHDA6, 13, 14 and 16 localized in both the cytosol and nucleus. Consistent with the predicted localization using bioinformatics programs, GmSRT4 and two members of HD2 family (GmHDT2 and GmHDT4) localized in the nucleus, mainly in the nucleolus (Fig. 3).

### Tissue and organ specific expression of *HDAC* genes in soybean

**Fig. 4:**
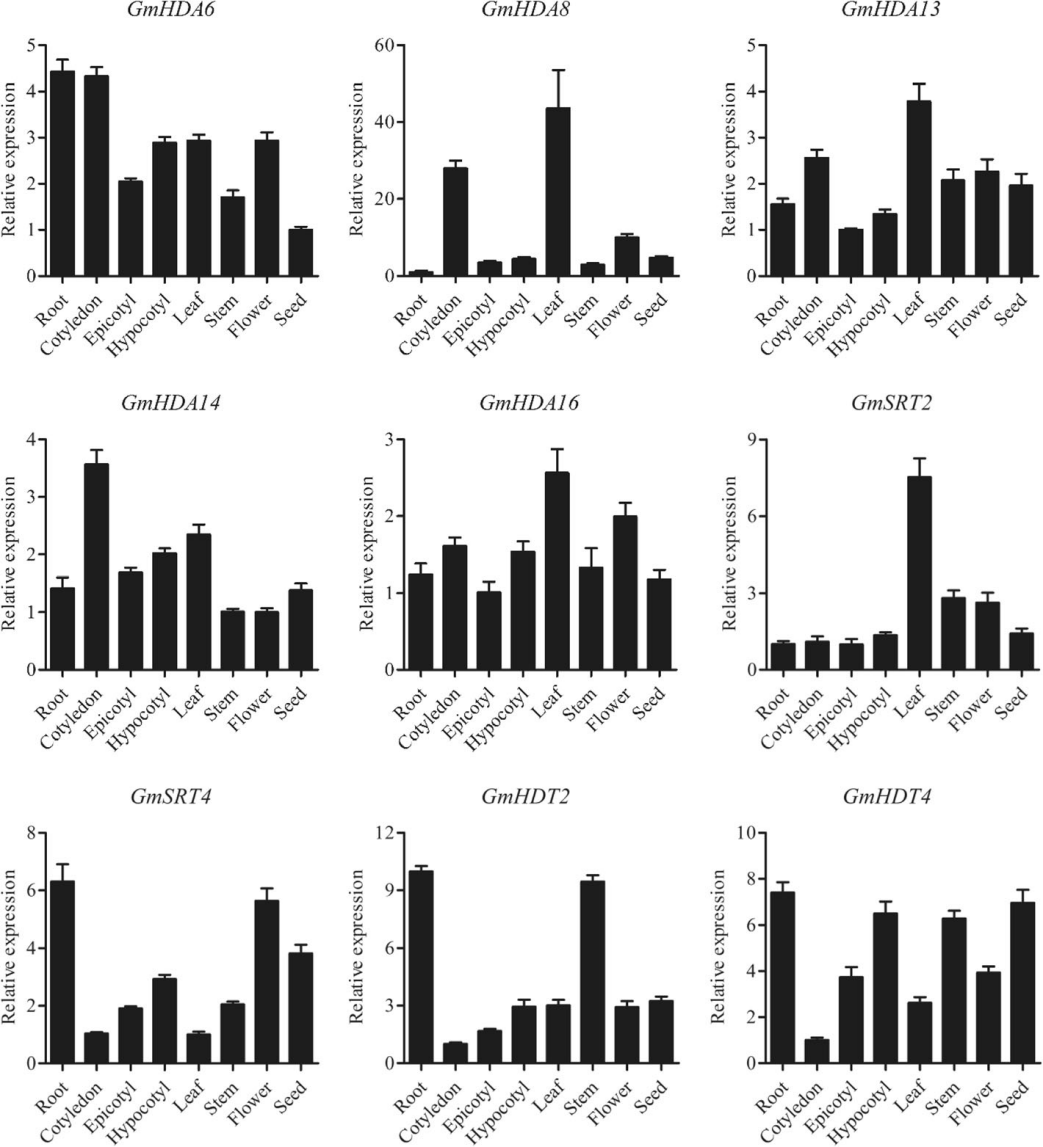
Tissue-specific expression patterns of *GmHDAC* genes. The x-axis represents different tissues or organs. The y-axis shows the gene expression levels after normalization to reference gene *GmCYP2*. Roots, cotyledons, epicotyls, and hypocotyls of 5-day-old plants and leaves, stems of 14-day-old plants, young flowers and seeds were collected for total RNA isolation. Quantitative RT-PCR was performed using gene-specific primers. Data are the mean ± SEM of three independent experiments

To investigate the tissue and organ specific expression profiles of *HDAC* genes in soybean, quantitative RT-PCR assays for nine representative *HDAC* genes from different families were conducted in different tissues and developmental stages. As shown in Fig. 4, *GmHDA6*, *GmHDA13*, *GmHDA14* and *GmHDA16* were ubiquitously expressed at high levels in most of the organs examined. *GmHDA8* showed high expression in cotyledon and leaf, as well as relatively low expression in other tissues. Specific high transcript accumulation of *GmSRT2* was observed in the leaves, while the expression in the other organs remained low. Unlike *GmSRT2*, *GmSRT4* mRNA levels were high in roots and flower tissues, moderate in hypocotyls and seeds, and lowest in the leaves. In addition, the *GmHDT2* transcripts were abundant in roots and stems, whilst relatively low in other tissues. Finally, *GmHDT4* was ubiquitously expressed at high levels in most of the organs examined, except for the cotyledons.

Soybean seeds are one of the most important agricultural commodities, being a prime source of oil, protein, and carbohydrate. To investigate the expression profiles of *GmHDAC* genes during soybean seed development and maturation, we obtained publicly available transcriptome data from the Soybase database (https://soybase.org/). As shown in Additional file 2: Fig. S1, nine *GmHDAC* genes displayed differential expression patterns in developing seeds. *GmHDA8*, *GmHDA14*, *GmHDT2* and *GmHDT4* were highly expressed throughout seed development. However, the expression of *GmSRT4* was only detected at the early stages of seed development, whereas *GmHDA13* was highly expressed during the middle stage. *GmHDA6* transcripts accumulated progressively early in developing seeds but their abundance decreased markedly during the seed maturation process. Additionally, the transcripts of *GmHDA16* were most abundant in 21 DAF (days after flowering) seeds but scarce in 28 DAF seeds (Additional file 2: Fig. S1).

### *GmHDACs* are involved in various abiotic stress responses

**Fig. 5:**
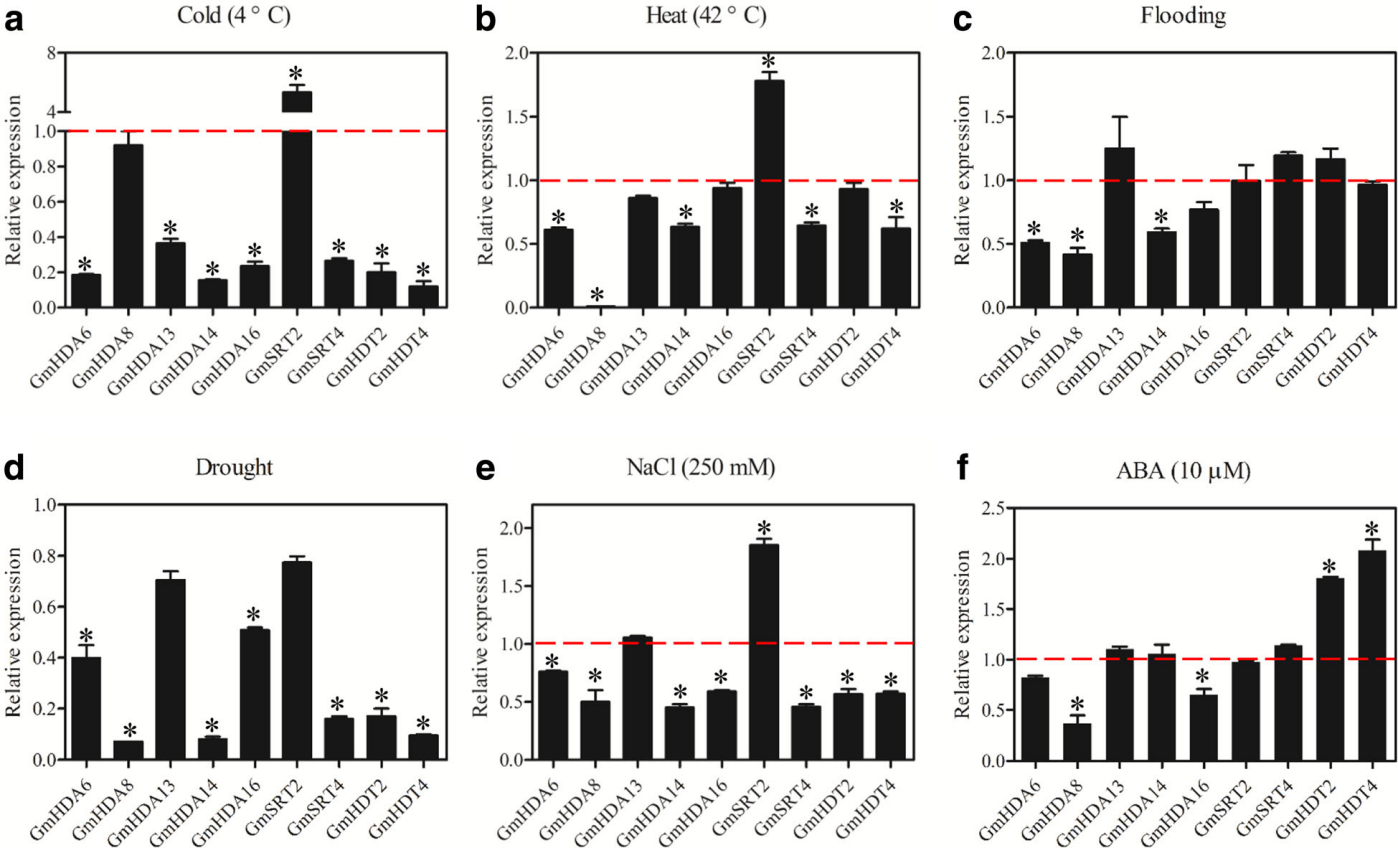
Expression profiles of *GmHDAC* genes under abiotic stresses. 10-day old plants were treated with cold (4 °C), heat (42 °C), flooding, air-drought, 250 mM NaCl and 10 μM ABA for 12 h and the leaves were harvested for qRT-PCR analysis of gene expression. The x-axis presents different genes. The y-axis shows expression levels relative to the control, which was set to 1.0. Data are the mean ± SEM of three independent experiments. *, *P*-value < 0.05, Student’s *t*-test

Evidence suggests that *HDACs* play important functions in plant response to various abiotic stresses. To study the potential roles of *GmHDACs* in abiotic stress responses, we performed expression analysis of *GmHDACs* under various abiotic stress conditions using quantitative RT-PCR. As shown in Fig. 5, our results demonstrated that *GmHDAC* genes significantly responded to various abiotic stress treatments. When exposed to cold, the expression of seven *GmHDAC* genes (*GmHDA6*, *13*, *14*, *16*, *GmSRT4*, *GmHDT2* and *GmHDT4*) was strongly repressed, while *GmSRT2* expression was significantly induced. On the other hand, *GmHDA8* was not significantly changed in response to low temperature (Fig. 5a). Following a heat shock treatment, five *GmHDAC* genes (*GmHDA6*, *8*, *14*, *GmSRT4* and *GmHDT4*) became down-regulated, *GmSRT2* was up-regulated, while the expression of *GmHDA13*, *16* and *GmHDT2* were unchanged (Fig. 5b). Notably, the accumulation level of the *GmHDA8* transcript was nearly completely suppressed under heat treatment (Fig. 5b). Furthermore, the differential expression patterns of the *GmHDAC* genes under flooding stress were observed. Four RPD3/HDA1 family genes, *GmHDA6*, *8*, *14* and *16* were down-regulated, while *GmHDA13*, *GmSRT4* and *GmHDT2* were slightly up-regulated and *GmSRT2* and *GmHDT4* did not respond to the flooding treatment (Fig. 5c). The expression levels of all nine *GmHDACs* examined decreased following a drought stress treatment with *GmHDA8*, *GmHDA14*, *GmSRT4*, *GmHDT2* and *GmHDT4* being the most impacted (Fig. 5d). Finally, high salt treatment significantly induced *GmSRT2* expression, but repressed the expression of *GmHDA6*, *8*, *14*, *16*, *GmSIR4*, *GmHDT2* and *GmHDT4*, and did not modulate the expression of *GmHDA13* (Fig. 5e).

**Fig. 6:**
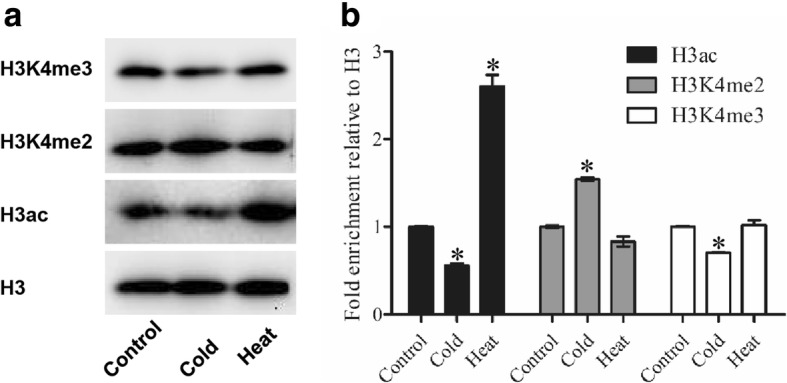
Levels of histone H3ac, H3K4me2, and H3K4me3 under cold and heat treatments. **a** Western blot showing the H3ac, H3K4me2, and H3K4me3 status in soybean leaves treated with cold and heat. 10-day-old seedlings were treated under cold (4 °C) and heat (42 °C) conditions for 12 h and the leaves were sampled for total protein extraction. **b** Quantification of western blot results. Signal intensities were measured using the ImageJ software and normalized to the loaded amount of H3. Values are expressed as fold change over control treatment. Shown is the mean ± SEM of three independent experiments. *, *P*-value < 0.05, Student’s *t*-test

Finally, we evaluated the responses of *GmHDACs* to abscisic acid (ABA) treatment, which is the most important stress-protective phytohormone. Two HD2 family genes (*GmHDT2* and *GmHDT4*) were induced and two RPD3/HDA1 family genes (*GmHDA8* and *GmHDA16*) were markedly suppressed by our ABA treatment. On the other hand, the expression of five other *HDAC* genes (*GmHDA6*, *13*, *14*, *GmSRT2* and *GmSRT4*) was only slightly modulated (Fig. 5f). In addition, status of H3 acetylation following our cold and heat treatments was analyzed. As shown in Fig. 6, the level of H3ac decreased under cold treatment, but increased after heat treatment. Interestingly, status of H3K4me2 and H3K4me3 were also modulated by the both cold and heat treatments. High levels of H3K4me2 and low levels of H3K4me3 were observed following our cold treatment. Meanwhile, the level of H3K4me2 was up-regulated in response to heat stress, while this treatment only had a mild effect on H3K4me3 levels (Fig. 6).

## Discussion

Studies have shown that HDACs play critical roles in multiple aspects of plant development and response to various environmental cues by regulating gene expression through histone deacetylation [4, 14, 15]. Genome-wide identification and characterization of HDACs have previously been reported in several plant species, including *Arabidopsis*, rice, grape (*Vitis vinifera*) and tomato (*Solanum lycopersicum*) etc. [10, 58–61]. However, the involvement of HDACs in the response to environmental cues has not been documented in soybean. In this study, 28 HDACs were identified and characterized in soybean, with respect to their tissue-specific expression profiles, subcellular localizations and abiotic stress responsive expression patterns. The 28 GmHDACs are divided into three families: RPD3/HDA1, SIR2 and HD2 and are unevenly distributed on 12 soybean chromosomes (Figs. 1 and 2). The number of *HDAC* gene in soybean is larger than those in *Arabidopsis*, rice and tomato and compared to other plant species, gene expansion of *HDACs* in soybean is evident [10, 58, 60, 61]. For example, *Arabidopsis*, rice and tomato only have two *SIR2* genes, while soybean contains four (Fig. 1a). Tandem amplification and segmental duplication of chromosomal regions are likely the main contributors for gene extension during evolution [56]. In total, 27 duplication pairs of *HDAC* genes were identified but no tandem gene sets were found (Fig. 2, Additional file 1: Table S1), indicating that segmental duplication events during evolution are most likely the major drivers of HDAC gene expansion in soybean.

In humans, RPD3 type Class I HDACs are localized exclusively in the nucleus, whereas class II HDACs are shuttled between the cytoplasm and the nucleus [62]. In *Arabidopsis*, previous studies demonstrated that RPD3 type Class II HDACs, HDA5, HDA8, and HDA14 are localized in the cytoplasm, whereas HDA15 is localized exclusively in the nucleus. In addition, AtHDA15 was shown to shuttle from the cytoplasm to the nucleus is response to light [63]. Moreover, RPD3 type Class I HDA6 and HDA19 also localized in the nucleus [45, 64, 65]. In this study, we found that GmHDA6, GmHDA13, GmHDA14 and GmHDA16 were localized in both the nucleus and cytoplasm (Fig. 3), suggesting a possible shuttling process between these compartments. Similar to the behavior of the *Arabidopsis* HD2 proteins [39], the two members of soybean HD2 family were localized in the nucleus (Fig. 3). SIR2 proteins were reported to occupy discrete subcellular compartments in plants. In addition, OsSRT1 appears to be mainly localized in the nucleus [66], while SlSRT2 localized in both the nucleus and cytoplasm [60] and OsSRT2 and AtSRT2 are found in the mitochondria [67]. In this study, we demonstrated that GmSRT4 localized in the nucleus (Fig. 3). Together, the different subcellular localization patterns of GmHDACs suggest that they might be differentially regulated and may have distinct roles in soybean. However, it is worth noting that the subcellular localization assays described in this study were conducted with *Arabidopsis* cells so it is entirely possible that soybean-specific interactions could affect the localization pattern in situ.

Increasing evidence suggests that the tissue-specificity and stress-responsiveness of *HDAC* genes play critical roles in plant development and environmental responses [14, 15, 68]. In our study, quantitative-RT PCR was employed to investigate the tissue-specific and stress-responsive expression patterns of nine typical *GmHDACs* (Figs. 4 and 5). *GmHDA13* was highly expressed in all tissues and developmental stages tested (Fig. 4), which is similar to its close homologous gene *AtHDA6*. Previous studies have shown that *AtHDA6* plays essential roles in various aspects of plant growth and development, such as leaf development and flowering, jasmonate and ethylene signaling, and abiotic stress responses [17–20, 48, 49, 65, 69, 70]. *GmHDA13* may thus have functions similar to those of *AtHDA6* considering their close evolutionary relationship and their similar expression patterns. *GmHDA8*, the closest homolog of *AtHDA14*, is preferentially expressed in cotyledons and leaves (Fig. 4), suggesting a potential role in regulating cotyledon and leaf development. Notably, the expression level of *GmSRT2* in leaves was much higher than in other organs, indicating that *GmSRT2* might play an important role in regulating leaf development (Fig. 4). In rice, *OsSRT1* was reported to regulate leaf senescence and cell death [66, 71], however, the functions of SIR2 proteins in plant growth and development remain largely unknown.

Microarray analysis demonstrated that several RPD3 type *HDACs* were repressed under high salt and drought treatments in rice [72]. Furthermore, the expression of *SlHDACs* was induced under salt, dehydration, and low/high temperature treatments, suggesting that *SlHDACs* might function in different stress responses in tomato [61]. In this study, we found these nine *GmHDACs* responded to various abiotic stress treatments, including low/high temperature, flooding, drought, NaCl and ABA (Fig. 5). Interestingly, the expression of most genes were repressed under the stress conditions examined, whilst *GmSRT2* was significantly induced both by cold and heat treatments (Fig. 5), indicating potentially distinct *HDAC* gene functions in response to different environmental cues. In *Arabidopsis*, ABA and NaCl treatments are known to repress the gene expression of *AtHD2A*, *AtHD2B*, *AtHD2C*, and *AtHD2D* [49]. AtHD2C interacts with AtHDA6 and regulates ABA-responsive gene expression by histone deacetylation [49]. Recently, Han et al. (2016) demonstrated that AtHD2D could confer tolerance to abiotic stresses, including drought, salt, and cold stresses in *Arabidopsis*. In addition, the transcription of HD2 type *HDACs* is modulated under salt, ABA, and PEG stress treatment in rice [73]. In the present study, *GmHDT2* and *GmHDT4* were down-regulated following a NaCl treatment and induced by the application of ABA, which is in support of previous findings (Fig. 5e, f). Furthermore, it has been shown that the expression of *HDACs* and the levels of histone H3K9ac, H4ac and H4K5ac were reduced by low temperature in maize [74]. Similarly, we demonstrated that cold treatment can repress the level of histone H3 acetylation (H3ac) in soybean while a heat treatment could increased H3ac (Fig. 6), indicating that histone acetylation may play pivotal roles in the plant responses to both cold and heat stress. Interestingly, the status of H3K4me2 and H3K4me3 were also modulated by the cold and heat treatments, indicating histone methyltransferases (HMTs) or histone demethylases (HDMs) may also be involved in these processes (Fig. 6).

## Conclusions

Our work identified 28 *HDAC* genes in soybean, which can be divided into RPD3/HDA1, SIR2, and HD2 families. Segmental duplication events during evolution were the major driver of *HDAC* gene expansion in soybean. Subcellular localization indicated that GmHDA6, GmHDA13, GmHDA14 and GmHDA16 were localized in both the nucleus and cytoplasm, while GmSRT4, GmHDT2 and GmHDT4 were found solely in the nucleus. Nine typical *GmHDAC* genes were differentially expressed in all tissues examined and all of them were stress-responsive. Interestingly, our results indicate that global histone acetylation and methylation levels were affected in response to cold and heat stress treatments in soybean, indicating that histone modifiers, such as HDACs might be involved in the response of this plant to abiotic stress. Further research is required to determine the function and molecular mechanisms of GmHDACs in plant responses to abiotic stress, which will provide tools for the improvement of soybean productivity.

## Methods

### Plant materials and treatments

Seedlings of soybean (*Glycine max*) were grown at 25 °C in a growth cabinet under a light intensity of 10,000 lx and a 16 h photoperiod. To investigate tissue or organ specific expression, roots, cotyledons, epicotyls, hypocotyls were harvested from 5-day-old seedlings, while leaves and stems were collected from 2-week-old seedlings. Flowers were collected when they were in full bloom. Seeds were collected 2 weeks after flowering. For the stress treatments, 10-day-old seedlings were exposed to different stress conditions and collected under the light period. Cold and heat stresses were performed by transferring soybean plants grown under control conditions into chambers set at 4 °C or 42 °C for the indicated period of time. The flooding treatment was performed by completely submerging seedlings under water at 25 °C. Dehydration was induced by removing plants from the pots and by placing them on filter paper at 25 °C. Soybean seedlings were exposed to salt stress by removing them from soil and by soaking them in a solution containing 250 mM NaCl. For ABA treatment, leaf tissues of the soybean plants were sprayed with 10 μM ABA solution. Each treatment was performed on five similar plants (same leaf number, leaf size and plant height) and seedlings without treatment were used as control. After exposure to these stresses for a period of 12 h, the plant tissues were harvested and immediately frozen in liquid nitrogen for further gene expression and protein immunoblotting experiments.

### Identification of *HDAC* genes in soybean

To identify potential HDACs in soybean, we used the protein sequences of AtHDACs as queries to run the BLASTP program against the soybean database (https://soybase.org/). After removing the duplicates from all the captured sequences, we initially retrieved the recognizable domains using BLAST-based NCBI conserved domain searches (https://www.ncbi.nlm.nih.gov/Structure/cdd/ wrpsb.cgi). We then further analyzed these domains using the HMMER-based Simple Modular Architecture Research Tool database (http://smart.embl-heidelberg.de/) and the Pfam software program (http://pfam.xfam.org/search).

### Phylogenetic tree construction

All the HDAC protein sequences from soybean and *Arabidopsis* were aligned using ClustalW, and the alignment was imported in MEGA5.0 for phylogenetic analysis. The phylogenetic trees were generated using the Maximum Parsimony method with partial deletion. The bootstrap value was assessed with 1000 replicates [75].

### Chromosomal localization and gene duplication analyses

The gene location and chromosome number for each soybean *HDAC* gene was retrieved from the Phytozome 12 *Glycine max* Wm82.a2.v1 database (www.phytozome.net). The Circos software was used to determine the chromosomal localization and generate the duplication image of the *GmHDAC* genes [76]. *GmHDAC* genes in duplicated genomic regions and the Ka/Ks values for the 27 duplicated gene pairs were obtained from Plant Genome Duplication Database (http://chibba.agtec.uga.edu/duplication/) to evaluate the contribution of tandem and segmental genome duplication to the expansion of the HDAC gene family over evolutionary time. Thus, homologous genes on the same duplicated chromosomal blocks were set as segmental duplication, while two paralogs physically close together were defined as tandem duplications. Ka/Ks values superior to 1 indicate positive selection while values below 1 and equal to 1 respectively indicate purifying selection and neural selection [57].

### RNA isolation and quantitative real-time RT-PCR

Total RNAs were isolated from the plant samples using the Hi-Pure Plant RNA Mini Kit (Magen, Guangzhou, China). The first-strand cDNA was generated using the TransScript One-Step gDNA Removal and cDNA synthesis SuperMix (TransGen, Beijing, China). The manufacturers’ instructions were followed in each case. Quantitative RT-PCR assays were performed on three biological replicates on a LightCycler 480 system (Roche, Basel, Switzerland) using TransStart Green qPCR SuperMix Kit (TransGen, Beijing, China). The reaction conditions were as follows: 95 °C for 1 min, followed by 50 cycles of 95 °C for 10 s, and 60 °C for 30 s. The soybean *CYP2* gene was used as the internal control, and the relative expression levels of genes were calculated using the 2^−ΔΔCT^ method [77]. Primer sequences are listed in Additional file 3: Table S2.

### Subcellular localization assays

The subcellular localizations of GmHDACs were first predicted using the pSORT web server (http://www.genscript.com/psort/wolf_psort.html) and confirmed by YFP-tagged transient expression assays in *Arabidopsis* suspension cultured cells. The full-length coding sequence of *GmHDACs* was introduced into pSAT6-EYFP-N1 to generate pSAT6-GmHDACs-EYFP, containing a GmHDACs-EYFP fusion construct under the control of the CaMV 35S promoter. The fusion constructs and nuclear localization marker NLS-mCherry were co-transfected into protoplast cells for in vivo protein targeting. The protoplast isolation and transient expression were conducted as described previously [78]. After transfection, the protoplasts were incubated at 22 °C for 12 h in the dark, and the distribution of the fusion protein was determined using a confocal fluorescence microscope.

### Protein immunoblotting

Soybean leaves sampled from control, cold (4 °C) and heat (42 °C) treated seedlings were ground to a powder in liquid nitrogen and mixed with 1 mL of ice-cold extraction buffer (0.1 M Tris-HCl, pH 8.0, 10% glycol, 3% SDS, 0.05% beta-mercaptoethanol). The samples were then boiled for 5 min, centrifuged at 13,000 rpm for 10 min and the supernatants collected as the total protein fractions. The protein were mixed with a loading dye and loaded on 12% polyacrylamide gels. After electrophoresis and transfer to a PVDF membrane, the samples were immunoblotted with the following commercial antibodies: anti-H3 (Millipore, 05–499), anti-H3ac (Millipore, 06–599), anti-H3K4me2 (Millipore, 07–030) and anti-H3K4me3 (Millipore, 04–745). The experiments were carried out three times and the Image J software was used to quantify the relative protein levels.

### Statistical analyses

A Student’s *t*-test (two tail, unpaired, equal variance) was used to determine the statistical significance of the differential transcripts abundance patterns and protein accumulation levels between treatments and their corresponding controls. Differential expression data were regarded as statistically significant and were marked by * only when passing the *t*-test with a *P*-value of < 0.05.

## Additional files

Additional file 1: **Table S1.** Segmental duplication events of soybean genes during evolution. (DOC 67 kb)

Additional file 2: **Figure S1.** Expression profiles of *GmHDAC* genes in developmental seeds. The transcript profiling data of soybean seeds was extracted from the publicly-available Soybase database (https://www.soybase.org/) for heatmap generation. The colors indicate expression intensity (red, high expression; black, low expression; grey, no expression). (JPG 538 kb)

Additional file 3: **Table S2.** Primers used in this study. (DOC 50 kb)

## Abbreviations

ABA: Abscisic acid
bp: base pair
CaMV: Cauliflower mosaic virus
cDNA: complementary deoxyribonucleicacid
DAF: days after flowering
gDNA: genomic deoxyribonucleicacid
kDa: kilo Dalton
NLS: nuclear localization signal
RNA: ribonucleicacid
RT-PCR: Real time-polymerase chain reaction
YFP: Yellow fluorescent protein
