# Supplementary material for: Characterization and subcellular localization of histone deacetylases and their roles in response to abiotic stresses in soybean
Source: BMC Plant Biol. 2018 Oct 11;18:226. doi: 10.1186/s12870-018-1454-7 (PMC6180487; doi:10.1186/s12870-018-1454-7)
Supplement: Supplementary file 1 — Table S1. Segmental duplication events of soybean genes during evolution. (DOC 67 kb) [file 12870_2018_1454_MOESM1_ESM.doc]

**Table S1.** Segmental duplication events of soybean genes during evolution.

| ID | LOCUS_1 | LOCUS_2 | BLOCK_NO | BLOCK_SCORE | E_VALUE | Ka | Ks | Ka/Ks |
| --- | --- | --- | --- | --- | --- | --- | --- | --- |
| 1 | Glyma.01G245100 | Glyma.11G000300 | 80 | 3177 | 3E-42 | 0.02 | 0.07 | 0.29 |
| 2 | Glyma.03G190700 | Glyma.11G189500 | 29 | 1064 | 3E-19 | 0.51 | 1.41 | 0.36 |
| 3 | Glyma.03G190700 | Glyma.12G181400 | 38 | 1402 | 1E-118 | 0.41 | 1.94 | 0.21 |
| 4 | Glyma.03G190700 | Glyma.12G084700 | 37 | 1351 | 2E-164 | 0.49 | 1.30 | 0.38 |
| 5 | Glyma.03G190700 | Glyma.13G319500 | 47 | 1715 | 8E-176 | 0.45 | 4.64 | 0.10 |
| 6 | Glyma.04G000200 | Glyma.06G000100 | 796 | 31732 | 0 | 0.01 | 0.1 | 0.10 |
| 7 | Glyma.04G187000 | Glyma.05G040600 | 118 | 4582 | 1E-96 | 0.12 | 0.55 | 0.22 |
| 8 | Glyma.04G187000 | Glyma.06G178700 | 711 | 28247 | 7E-172 | 0.12 | 0.30 | 0.40 |
| 9 | Glyma.04G187000 | Glyma.17G085700 | 74 | 2886 | 0 | 0.12 | 0.54 | 0.22 |
| 10 | Glyma.04G210000 | Glyma.06G156000 | 711 | 28247 | 7E-172 | 0.03 | 0.13 | 0.23 |
| 11 | Glyma.05G012900 | Glyma.17G120900 | 62 | 2474 | 0 | 0.04 | 0.23 | 0.17 |
| 12 | Glyma.05G021400 | Glyma.17G078000 | 64 | 2547 | 0 | 0.04 | 0.13 | 0.31 |
| 13 | Glyma.05G040600 | Glyma.06G178700 | 149 | 5786 | 1E-170 | 0.20 | 0.60 | 0.33 |
| 14 | Glyma.05G040600 | Glyma.17G085700 | 141 | 5624 | 0 | 0.01 | 0.12 | 0.08 |
| 15 | Glyma.06G178700 | Glyma.17G085700 | 84 | 3300 | 2E-50 | 0.26 | 0.62 | 0.42 |
| 16 | Glyma.08G330200 | Glyma.18G076300 | 326 | 12791 | 2E-72 | 0.01 | 0.06 | 0.17 |
| 17 | Glyma.11G187800 | Glyma.12G086700 | 121 | 4808 | 6E-18 | 0.01 | 0.08 | 0.13 |
| 18 | Glyma.11G189500 | Glyma.12G084700 | 121 | 4808 | 6E-18 | 0.03 | 0.20 | 0.15 |
| 19 | Glyma.11G189500 | Glyma.12G181400 | 61 | 2380 | 8E-12 | 0.24 | 0.65 | 0.37 |
| 20 | Glyma.11G189500 | Glyma.13G319500 | 59 | 2305 | 0 | 0.50 | 1.17 | 0.43 |
| 21 | Glyma.11G189500 | Glyma.19G191000 | 11 | 399 | 6E-73 | 0.51 | 1.32 | 0.39 |
| 22 | Glyma.12G084700 | Glyma.12G181400 | 150 | 5805 | 0 | 0.30 | 0.54 | 0.56 |
| 23 | Glyma.12G084700 | Glyma.13G319500 | 150 | 5835 | 9E-179 | 0.30 | 0.65 | 0.46 |
| 24 | Glyma.12G084700 | Glyma.19G191000 | 33 | 1197 | 2E-124 | 0.53 | 1.26 | 0.42 |
| 25 | Glyma.12G181400 | Glyma.13G319500 | 438 | 17413 | 0 | 0.08 | 0.23 | 0.35 |
| 26 | Glyma.12G181400 | Glyma.19G191000 | 35 | 1292 | 9E-124 | 0.43 | 2.04 | 0.21 |
| 27 | Glyma.13G319500 | Glyma.19G191000 | 46 | 1689 | 3E-50 | 0.46 | 1.92 | 0.24 |
