# Supplementary figures and images for: Characterization and subcellular localization of histone deacetylases and their roles in response to abiotic stresses in soybean
Source: BMC Plant Biol. 2018 Oct 11;18:226. doi: 10.1186/s12870-018-1454-7 (PMC6180487; doi:10.1186/s12870-018-1454-7)

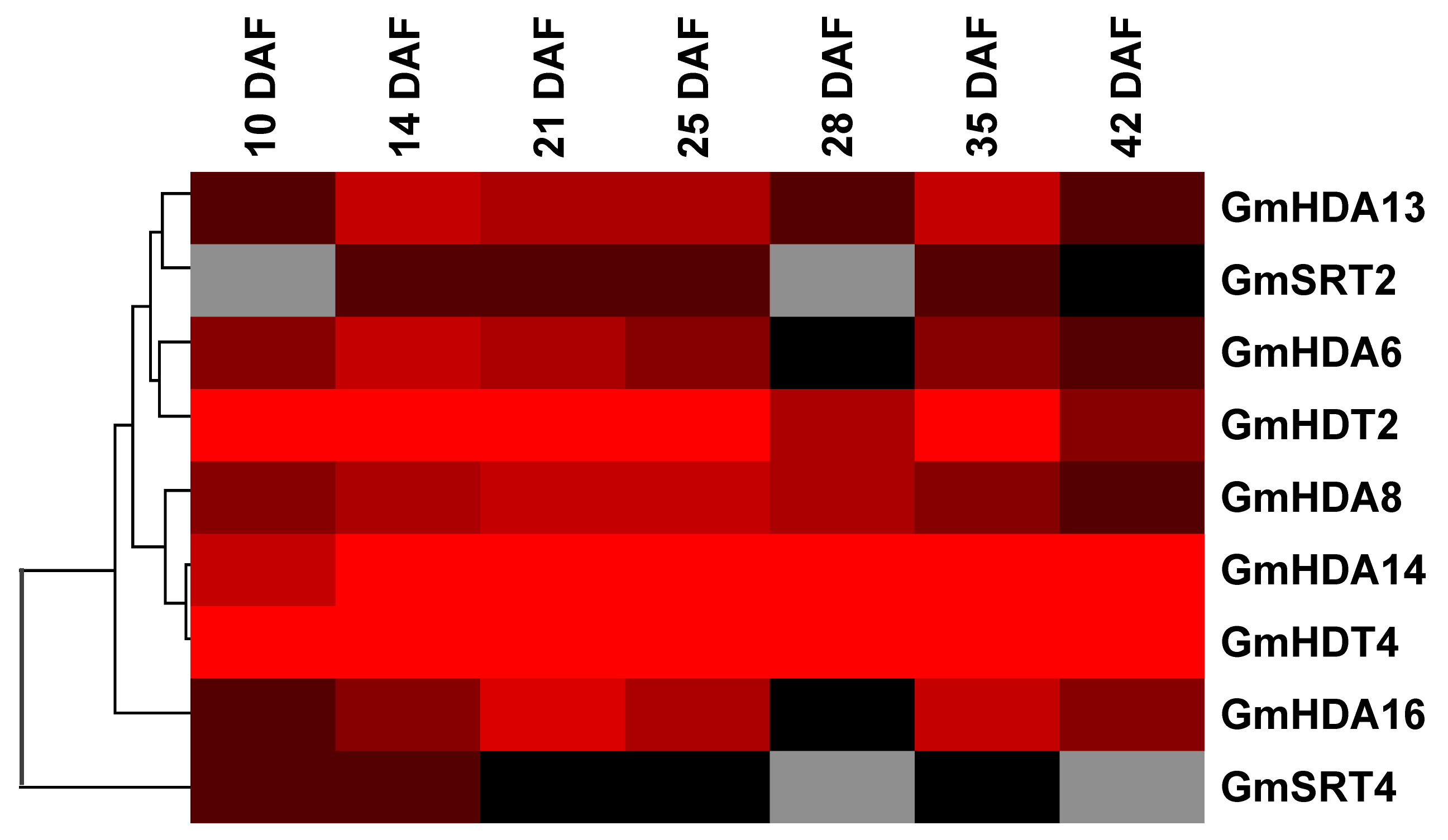

Supplement: Supplementary file 2 — Figure S1. Expression profiles of GmHDAC genes in developmental seeds. The transcript profiling data of soybean seeds was extracted from the publicly-available Soybase database (https://www.soybase.org/) for heatmap generation. The colors indicate expression intensity (red, high expression; black, low expression; grey, no expression). (JPG 538 kb) [file 12870_2018_1454_MOESM2_ESM.jpg]
