# Supplementary material for: Characterization and subcellular localization of histone deacetylases and their roles in response to abiotic stresses in soybean
Source: BMC Plant Biol. 2018 Oct 11;18:226. doi: 10.1186/s12870-018-1454-7 (PMC6180487; doi:10.1186/s12870-018-1454-7)
Supplement: Supplementary file 3 — Table S2. Primers used in this study. (DOC 50 kb) [file 12870_2018_1454_MOESM3_ESM.doc]

**Table S2.** Primers used in this study.

| **Gene ID** | **Gene Name** | **Primer** | **Sequence** |
| --- | --- | --- | --- |
| Glyma.05g021400 | *GmHDA6* | GmHDA6-qF | GCACCACCTCCGCATACTCTTGT |
|  |  | GmHDA6-qR | TGACAATTCAGATCGCCAAGTACTA |
|  |  | GmHDA6-YFP-F | GCTCAAGCTTCGAATTCATGGAACGTGAAAACGACAACA |
|  |  | GmHDA6-YFP-R | ACCATCAGGATCCCGGGCAATGATTTAGCAGCACCTTCT |
| Glyma.05g192600 | *GmHDA8* | GmHDA8-qF | GGTTATGGATCAAGCTGTGGAGAAA |
|  |  | GmHDA8-qR | AACTGCGTGATGTCCTGGTGGTC |
| Glyma.12g086700 | *GmHDA13* | GmHDA13-qF | GGCGATGCTAAGGAAATAGGAGA |
|  |  | GmHDA13-qR | CTCCAGTGACCAGCAATGGCAAA |
|  |  | GmHDA13-YFP-F | GCTCAAGCTTCGAATTCATGCGCTCCAAGGACAGAATCG |
|  |  | GmHDA13-YFP-R | ACCATCAGGATCCCGGGTGAAATATTCATTTGATCATTG |
| Glyma.12g188200 | *GmHDA14* | GmHDA14-qF | CCTATCTCCCCTTACATTTCTTGGC |
|  |  | GmHDA14-qR | GATAGTGTAGTCCCAGCAGCAAG |
|  |  | GmHDA14-YFP-F | GCTCAAGCTTCGAATTCATGGCTGATGCAGGTGCAGAAG |
|  |  | GmHDA14-YFP-R | ACCATCAGGATCCCGGGGCACACTTTATCTTTTTGATAA |
| Glyma.17g085700 | *GmHDA16* | GmHDA16-qF | TGTGGAGCTGATTCATTGTCTGGTG |
|  |  | GmHDA16-qR | CACTCCTACTGCCACTGCTGTCT |
|  |  | GmHDA16-YFP-F | GCTCAAGCTTCGAATTCATGGGAATGGAAGAGGAGAGTA |
|  |  | GmHDA16-YFP-R | ACCATCAGGATCCCGGGACTACAAGATGATGGATGCTCT |
| Glyma.06g156000 | *GmSRT2* | GmSRT2-qF | ACTGATGGAAAGACCTCAACTAATA |
|  |  | GmSRT2-qR | CATTAAGTCGATTCGGCCAGCTCTA |
| [Glyma.18g076300](https://soybase.org/sbt/search/search_results.php?category=FeatureName&version=Glyma2.0&search_term=Glyma.18g076300) | *GmSRT4* | GmSRT4-qF | ATTTCGACCCATCCCATGTTTTGCG |
|  |  | GmSRT4-qR | TCAGGCAGGGCTTTACCTTCACG |
|  |  | GmSRT4-YFP-F | GCTCAAGCTTCGAATTCATGTCTCTGGGGTATGCCGAGA |
|  |  | GmSIR4-YFP-R | ACCATCAGGATCCCGGGCGTATGACCCCTAATATTGTCT |
| Glyma.11g189500 | *GmHDT2* | GmHDT2-qF | ACTAACTGGCGGCAACAATGCTTCT |
|  |  | GmHDT2-qR | TAGCAGGGGTCTCTTCATCACTCTC |
|  |  | GmHDT2-YFP-F | GCTCAAGCTTCGAATTCATGGATGCACCAATGGAGTTTT |
|  |  | GmHDT2-YFP-R | ACCATCAGGATCCCGGGCTGACCACCATGCTTAGCCTTC |
| Glyma.12g181400 | *GmHDT4* | GmHDT4-qF | GAAGGCAGATTAGGAAAGAAGAG |
|  |  | GmHDT4-qR | CAGGGTGAGGAGTTGCTGTATGT |
|  |  | GmHDT4-YFP-F | GCTCAAGCTTCGAATTCATGGAGTTTTGGGGTGCCGAGG |
|  |  | GmHDT4-YFP-R | ACCATCAGGATCCCGGGCTGACCACCATGCTTTGCCTTG |
| Glyma.12g024700 | *GmCYP2* | GmCYP2-qF | CGGGACCAGTGTGCTTCTTCA |
|  |  | GmCYP2-qR | CCCCTCCACTACAAAGGCTCG |
